# The Influence of Running Cadence on Biomechanics and Injury Prevention: A Systematic Review

**DOI:** 10.7759/cureus.90322

**Published:** 2025-08-17

**Authors:** Inês Figueiredo, Miguel Reis e Silva, José Eduardo Sousa

**Affiliations:** 1 Physical Medicine and Rehabilitation, Centro de Reabilitação de Alcoitão, Lisbon, PRT; 2 Health and Performance, Sport Lisboa e Benfica, Lisbon, PRT; 3 Physical Medicine and Rehabilitation, Centro de Medicina de Reabilitação da Região Centro - Rovisco Pais, Tocha, PRT

**Keywords:** biomechanics, cadence, gait retraining, overuse injury, running

## Abstract

Running is a widely practiced physical activity with well-established health benefits. However, it is frequently associated with overuse musculoskeletal injuries. Among the modifiable biomechanical variables, increasing cadence, defined as the number of steps per minute, has emerged as a promising target for reducing injury risk by altering impact forces and load distribution. The aim of this review was to systematically evaluate and critically appraise the scientific evidence on the effects of running cadence modification on biomechanics and injury prevention in adult runners. Following PRISMA (Preferred Reporting Items for Systematic Reviews and Meta-Analyses) guidelines, a systematic search was conducted in PubMed, Scopus, and Web of Science (2009-2025). Eighteen studies were included, encompassing randomized controlled trials, quasi-experimental studies, cross-sectional analyses, and systematic reviews. Cadence was the main variable examined in relation to biomechanical or clinical outcomes. Data were extracted by three independent reviewers, and a narrative synthesis was performed due to methodological heterogeneity.

A moderate increase in cadence (typically 5-10%) led to consistent biomechanical improvements, including reduced vertical ground reaction forces, lower loading rates, shorter stride length, and improved lower limb alignment. These adaptations were associated with reduced stress on the tibia, knee, and hip joints. Importantly, cadence modification did not negatively affect metabolic cost and, in some cases, enhanced running economy. Auditory cueing strategies facilitated adherence, and evidence suggested a preventive effect on injuries such as patellofemoral pain and tibial stress fractures. Cadence retraining appears to be a low-cost, accessible, and effective strategy to optimize running biomechanics and potentially reduce the incidence of overuse injuries. Further high-quality prospective studies are needed to confirm its long-term clinical and performance-related benefits.

## Introduction and background

Running is a globally practiced form of physical activity, valued not only for its accessibility but also for its well-established cardiovascular, metabolic, and psychological benefits. Despite these advantages, running is associated with a high incidence of musculoskeletal injuries, primarily due to the repetitive nature and high-impact forces generated during the running gait cycle [1]. Lower extremity running-related injuries occur in an estimated 56% of recreational runners [1] (defined as individuals who run regularly for health, leisure, or personal goals without competing at an elite level), underscoring the need for effective preventive strategies and a deeper understanding of the biomechanical risk factors involved.

The most common overuse injuries in runners include patellofemoral pain syndrome, medial tibial stress syndrome (MTSS), Achilles tendinopathy, plantar fasciitis, iliotibial band syndrome, and tibial stress fractures. In elite and competitive runners, tendinopathies and bone stress injuries tend to be more prevalent, often exacerbated by high training loads, poor joint alignment, inadequate recovery, and intense competition schedules.

These injuries result from the complex interaction between intrinsic and extrinsic risk factors. Intrinsic factors include anatomical alignment (e.g., excessive hip internal rotation, foot pronation), muscle imbalances, sex, age, and previous injury history. Extrinsic factors include training errors (e.g., sudden volume increases), inappropriate footwear, running surface, and insufficient recovery periods. Among these, some risk factors are modifiable (e.g., cadence, stride length, load management), whereas others are non-modifiable (e.g., age, sex, limb alignment).

The biomechanics of running, which includes parameters such as stride pattern (the length and rhythm of each step), cadence, ground contact time, joint alignment (the relative positioning of joints during movement), and ground reaction forces (the force exerted by the ground back on the body with each foot strike), directly influences the loads imposed on osteoarticular and musculoskeletal structures. Typical cadence values in recreational runners range from 150 to 170 steps per minute, while elite runners often exceed 180 steps per minute. Ground contact time during running is usually between 200 and 300 milliseconds, and stride length tends to decrease as cadence increases. These parameters are interdependent and play a key role in modulating impact forces and joint loading. Among them, cadence (defined as the number of steps per minute) has been identified as one of the most influential and modifiable factors for optimizing running mechanics and reducing injury risk [2,3].

Despite this growing interest, there is still a lack of consensus regarding the extent to which cadence modifications translate into clinically meaningful benefits. Many studies report surrogate biomechanical outcomes rather than long-term injury rates, and methodological heterogeneity limits generalization. This reflects a clear gap in the literature, particularly regarding the sustainability of cadence interventions and their applicability across different runner profiles.

Therefore, this systematic review aims to synthesize and critically appraise current evidence on the influence of cadence modification on running biomechanics and injury prevention in adult runners. It explores biomechanical adaptations associated with cadence changes, their potential impact on injury mechanisms, and the feasibility of implementing cadence retraining in clinical and athletic settings.

## Review

Methodology

**Figure 1:**
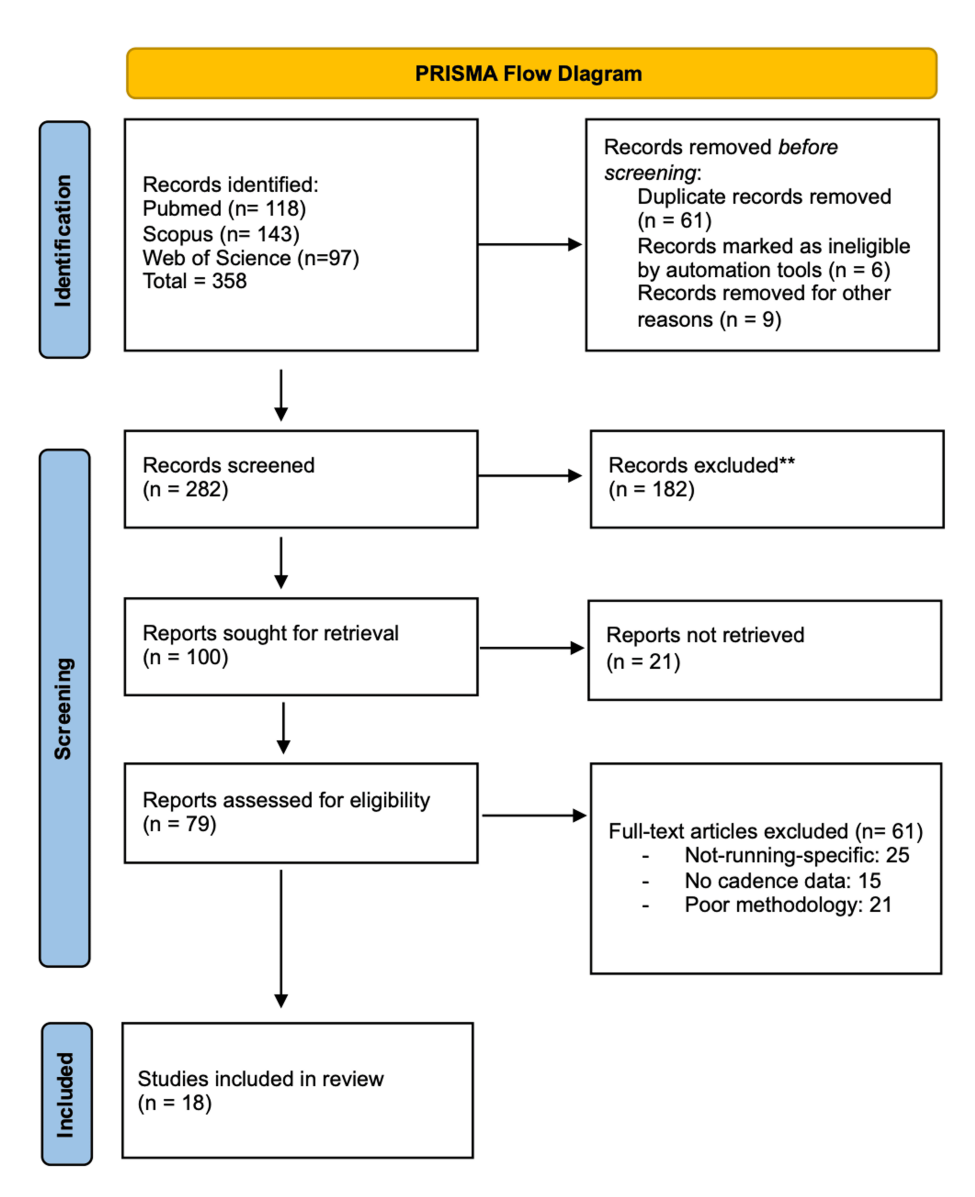
PRISMA flow diagram. PRISMA: Preferred Reporting Items for Systematic Reviews and Meta-Analyses.

This systematic review was developed in accordance with recognized methodological guidelines for reviews in the field of health and sports sciences, with the aim of gathering and critically analyzing the existing scientific literature on the influence of cadence on running biomechanics and injury prevention. To ensure transparency, reproducibility, and reliability in the process of selecting and analyzing the included studies, the PRISMA (Preferred Reporting Items for Systematic Reviews and Meta-Analyses) statement was followed. Although a formal meta-analysis was not performed due to the heterogeneity of study designs and outcomes, the procedures for identification, screening, eligibility, and inclusion of articles were conducted in line with internationally recognized good practices for systematic reviews and are detailed in the PRISMA flow diagram (Figure 1).

Searches were carried out in international databases (PubMed, Scopus, and Web of Science), covering publications from 2009 to March 2025. The final literature search was conducted in April 2025. In the PubMed database, the MeSH terms used were “running,” “cadence”, “stride length”, “injury”, “injury prevention”, and “biomechanics”. Reference lists of key articles (including already published reviews) were also consulted to identify additional relevant studies. There were no language restrictions, although all included studies were published in English, the dominant language in the scientific literature on this subject.

Inclusion and exclusion criteria

Studies were included if they met the following criteria: participants were adult runners (either recreational or competitive), regardless of sex or level of experience. The studies had to analyze running cadence as a primary variable, either in a natural context or through induced modulation methods such as metronome, music, or visual feedback. Eligible studies needed to assess outcomes related to running biomechanics and injury risk, including variables such as impact forces, joint alignment, or the incidence of running-related injuries. The types of studies considered included clinical trials (randomized or quasi-experimental), cross-sectional or longitudinal observational studies, technological validation studies, as well as systematic or narrative reviews synthesizing relevant scientific evidence. Only articles published in peer-reviewed scientific journals or chapters in recognized academic books were considered.

Studies were excluded if they were case reports or case series without a comparison group, if they involved non-running populations, or if they included pediatric or geriatric individuals with low levels of running activity. Articles were also excluded if their methodology was not clearly described or if their analysis of cadence was unrelated to any biomechanical or injury-related outcome.

Study selection and data extraction

The selection process involved two stages: an initial screening of titles and abstracts, followed by a full-text assessment of potentially eligible articles. All three authors independently performed study selection and data extraction, with discrepancies resolved by consensus to ensure rigorous and consistent inclusion of studies.

For each study included, the following data were extracted: authors, year of publication, type of study, sample characteristics, description of the intervention or cadence analysis method, biomechanical variables analyzed, clinical outcomes (if applicable), and main quantitative and qualitative results.

Given the heterogeneity of study designs, no formal meta-analysis was performed. However, a structured quality appraisal was conducted to ensure the reliability and relevance of the included studies.

Quality assessment

The methodological quality of the studies included in this systematic review was assessed using validated tools, selected according to the design of each study. The specific quality assessment tool applied to each individual study is presented in Table 1.

For experimental and quasi-experimental studies, the NIH Quality Assessment Tool for Observational Cohort and Cross-Sectional Studies was employed. Developed by the National Institutes of Health, this tool evaluates internal validity by examining domains such as sample selection, blinding procedures, and statistical analysis methods [4].

Cross-sectional studies were appraised using the AXIS Tool, which provides a structured framework to evaluate reporting clarity, study design, and potential sources of bias [5].

To evaluate observational cohort and case-control studies, the Newcastle-Ottawa Scale (NOS) was applied. This tool assesses study quality across three domains: selection of participants, comparability of study groups, and ascertainment of the outcome. It is widely recommended for use in systematic reviews that include observational studies [6].

For randomized clinical trials within the domain of physical therapy and biomechanics, the PEDro Scale was used. This 11-item scale evaluates methodological rigor, focusing on aspects such as randomization, allocation concealment, blinding, and adequacy of statistical reporting [7].

Systematic reviews and meta-analyses included in this review were assessed using the AMSTAR 2 tool (A MeaSurement Tool to Assess Systematic Reviews 2). This instrument evaluates 16 methodological aspects, including protocol registration, comprehensiveness of the literature search, assessment of risk of bias, and appropriateness of meta-analytic methods [8].

The overall quality ratings of the included studies, categorized as high, moderate, or low, are presented in Table 1, based on the scoring criteria of each respective tool.

Results

After applying the inclusion and exclusion criteria, a total of 18 studies were selected, representing a range of methodological designs, including controlled clinical trials, cross-sectional laboratory studies, systematic reviews with meta-analysis, and technological validation studies. These studies involved populations of recreational and competitive runners, with sample sizes ranging from 7 to over 1000 participants.

Table 1 summarizes the included studies, presenting information on author/year, type of study, sample, main measures, relevant findings, quality assessment tool, and quality rating/score.

**Table 1:** Overview of the studies included.

Author (Year)	Study Type	Sample	Main Measures	Relevant Findings	Assessment Tool	Quality Rating / Score
Musgjerd et al. (2021) [1]	Quasi-experimental study	13 recreational runners	Cadence (baseline, +5%, +10%), impact force outdoors	Increasing cadence by 5% and 10% significantly reduced impact force. Applying the increase in cadence in a real environment (outside the laboratory) proved to be effective and feasible	NIH Tool	Moderate
Heiderscheit et al. (2011) [2]	Laboratory study (crossover)	45 healthy recreational runners	3D kinematics and kinetics (ground reaction forces, joint moments) at preferred cadence vs. +5% and +10% of cadence	Increasing cadence by 5% significantly reduced hip and knee loading (lower thigh adduction moment, lower impact force); +10% accentuated these reductions. Shorter strides have also been shown to reduce stride length (avoiding overstriding) without substantially increasing energy costs	NIH Tool	Moderate
Lenhart et al. (2014) [3]	Experimental study	30 healthy runners	3D biomechanical and EMG analysis during running with an increased cadence (5% and 10%)	Increases in cadence significantly reduced joint loads in the knee and hip, suggesting that there is a protective biomechanical effect	NIH Tool	Moderate
Nijs et al. (2020) [9]	Laboratory study (secondary data analysis)	16 recreational runners	Vertical impact force and load rate measured on instrumented treadmill, under conditions: normal cadence vs. +7.5% vs. +15%; with and without acoustic stimulus (metronome)	Increasing cadence significantly reduced impact force and vertical load rate in all runners. The use of a metronome (acoustic pacing) itself did not negatively affect the forces - in other words, the reductions observed came from the increase in cadence, with no adverse effect from the metronome	NIH Tool	Moderate
Peterson et al. (2024) [10]	Quasi-experimental study (laboratory)	10 asymptomatic recreational runners	Dynamic knee valgus angle measured in 3D during treadmill running at usual cadence vs. 110% of that cadence	A 10% increase in cadence significantly reduces the dynamic knee valgus angle by ~2° (on average). There was also a concomitant reduction in hip adduction during the support phase. These biomechanical adjustments may relieve patellofemoral pressure, suggesting benefit in the prevention/treatment of patellofemoral syndrome	AXIS Tool	High
Anderson et al. (2022) [11]	Systematic review and meta-analysis	37 studies, runners	Running cadence, injury prevention, and biomechanics	Increased cadence reduces injury risk and improves biomechanics	Newcastle-Ottawa Scale (NOS)	High
Brake et al. (2023) [12]	Randomized multiple baseline design	5 recreational runners (4 men, 1 woman), aged 22–43 years, injury-free at time of testing	Running cadence, stride length, vertical center of mass displacement, ground contact time, and perceived exertion	The use of music with a beat frequency effectively increased running cadence in all participants. This was accompanied by biomechanical changes such as decreased stride length and vertical oscillation, which are associated with reduced impact forces. Music-guided cadence manipulation is a feasible and effective strategy to alter running biomechanics and may help reduce injury risk in recreational runners	PEDro Scale	High
Van Hooren et al. (2024) [13]	Systematic review with meta-analysis	51 studies; 1115 runners included	Cadence, stride length, ground contact time, oxygen consumption (VO2)	A higher cadence correlates with lower energy consumption (r = -0.20). It has been shown to have a positive impact on running economy, and that changes in cadence do not compromise physical performance	AMSTAR 2	High
Hollander et al. (2019) [14]	Randomized clinical trial	60 runners (53 completed)	Cadence, impact force, load rate	Running barefoot has been shown to increase cadence and reduce impact initially. But the benefits were not maintained without continuous feedback (after habituation of 8 weeks)	NIH Tool	Moderate
Luedke et al. (2016) [15]	Prospective cohort (12 weeks)	68 high school cross-country runners (16±1 years)	Preferred cadence (steps/min) measured at the beginning of the season; incidence of injuries during the season	Runners with low cadences (≤~166 spm) had a ~6-7 times higher risk of tibial injury (“shin splints”/stress fracture) compared to athletes with higher cadences (≥~178 spm). Associations with anterior knee pain were less consistent, but the trend was towards a lower incidence with higher cadences	Newcastle-Ottawa Scale (NOS)	High
Stockland et al. (2019) [16]	Experimental cross-sectional study	39 healthy runners	Self-selected cadence; % weight bearing	Cadence decreases with lower body weight; Most were able to maintain or increase with instruction	NIH Tool	Moderate
Nicolas-Peyrot et al. (2025) [17]	Quasi-experimental	23 recreational runners	Cadence modification, footwear, orthoses, plantar pressure	A moderate increase in cadence reduced rearfoot impact forces and shortened stride length, suggesting beneficial biomechanical changes in recreational runners	Newcastle-Ottawa Scale (NOS)	High
Menéndez et al. (2020) [18]	Systematic review	11 observational studies	Risk factors for Medial Tibial Stress Syndrome (MTSS), interventions, and cadence	Modifying running technique, including increasing cadence, may help reduce tibial load and prevent MTSS in recreational runners	Newcastle-Ottawa Scale (NOS)	Moderate
Chan et al. (2018) [19]	Randomized controlled trial	320 novice runners	Vertical loading rates, injury incidence	62% reduction in injury risk after 12 months	PEDro Scale	High
Allen et al. (2016) [20]	Experimental study (pre-post intervention)	20 long-distance runners (heel-strikers)	Support pattern (rearfoot vs midfoot/forefoot) before and after training with cadence +10%	An 8-week training program with an increase in cadence of ~10% was carried out, which converted most of the runners from a heel-strike support pattern to a midfoot or forefoot pattern. This change is associated with reductions in the initial impact forces of foot contact	NIH Tool	Moderate
Schubert et al. (2014) [21]	Systematic review	35 observational studies (n≈600)	Review of studies varying cadence and measuring effects on mechanics. Qualitative meta-analysis of relationships between step frequency and biomechanical variables	Concluded that increasing step frequency (cadence) tends to result in a shorter stride, shorter ground contact time, and less vertical displacement, associated with reductions in lower limb joint loads. Consistent findings include a decrease in peak ground reaction force and hip adduction moment with higher cadences	AMSTAR 2	Moderate
de Souza Júnior et al. (2024) [22]	Randomized controlled trial	60 runners with patellofemoral pain	Pain, function, lower limb kinematics	Gait retraining programs improve pain and function in runners, with better lower limb alignment	Newcastle-Ottawa Scale (NOS)	High
Chumanov et al. (2012) [23]	Randomized controlled trial	45 healthy runners	Muscle activation, running cadence	Increased cadence improved muscle activation during the late swing phase, reduced peak hip adduction, and had therapeutic benefits for knee pain	Newcastle-Ottawa Scale (NOS)	High

Cadence and biomechanical adaptations

The studies analyzed show that a moderate increase in cadence, generally between 5% and 10% [1] above the spontaneous cadence, promotes significant biomechanical changes during running. Among the main effects is a reduction in ground reaction forces and vertical loading rate, attributed to a shorter stride and the placement of the foot closer to the body’s center of mass. This adaptation reduces the phenomenon of overstriding and the vertical oscillation of the body, mitigating joint impact. Studies such as those by Nijs et al. (2020) [9] and Heiderscheit et al. (2011) [2] have shown substantial reductions in peak impact, with decreases of approximately 20% at the knee level. These adaptations are particularly relevant for runners prone to patellofemoral pain or tibial stress injuries.

In tightly controlled treadmill experiments, with running velocity held constant, these effects were observed independent of speed (e.g., Heiderscheit et al. [2]); in contrast, some field or quasi-experimental protocols allowed self-selected speed (e.g., Musgjerd et al. [1]), which may partly confound the isolated contribution of cadence.

Kinematic improvements and joint alignment

Several studies reported improvements in lower-limb alignment with increased cadence. Typical findings included reduced dynamic knee valgus and decreased hip adduction during stance, together with shorter ground contact time.

Peterson et al. (2024) [10] reported a reduction of approximately two degrees in dynamic valgus with a 10% cadence increase under laboratory conditions, while other authors have identified shorter contact time with the ground, favoring a more balanced distribution of joint loads. Speed was kept constant in most treadmill designs, whereas velocity control was not always explicitly stated in smaller experimental reports. Collectively, these kinematic shifts are consistent with a more favorable load distribution across the knee and hip.

Impact forces and joint load distribution

Anderson et al. (2022) [11] emphasized that increasing running cadence can significantly reduce the risk of injury by improving biomechanics. Their systematic review and meta-analysis found that a higher cadence decreased impact forces and improved the distribution of joint loads, which may help in injury prevention and enhance running efficiency. Biomechanical models suggest that these adaptations help reduce the risk of stress fractures, despite the increase in the total number of steps. Many of the primary studies contributing to that review standardized running speed during cadence manipulation, strengthening internal validity. Still, a minority of quasi-experimental studies did not report strict velocity control, indicating some between-study heterogeneity in exposure definition.

Running economy and metabolic efficiency

As far as running economy is concerned, the data point to no negative impact, and small gains in locomotor efficiency are even suggested. Brake et al. (2022) [12] and Van Hooren et al. (2024) [13] reported no significant changes in heart rate or oxygen consumption, reinforcing the metabolic feasibility of this intervention. In some cases, running economy even improved slightly, indicating that biomechanical gains can be achieved without compromising energy efficiency. Evidence indicates that moderate cadence increases do not adversely affect metabolic cost when running speed is held constant.

Sustainability of cadence modifications

These modifications can be implemented in a simple and cost-effective manner using tools such as metronomes, tempo-based music, or real-time auditory and visual feedback, which enhance adherence both in clinical settings and in self-guided training. Maintaining these biomechanical adaptations over time requires consistent practice and reinforcement of new motor patterns.

Without continued feedback, partial regression to baseline has been reported (Hollander et al. [14]). Interventions that used auditory cueing (metronome or beat-synchronous music) improved adherence and maintenance (te Brake et al. [12]); most longitudinal follow-ups tested at constant treadmill speed, although some rehabilitation-context studies allowed self-selected speeds, potentially increasing measurement variability.

Long-term adherence remains a challenge, and evidence suggests that without structured follow-up, the benefits of cadence retraining may diminish over time. Therefore, strategies for monitoring and reinforcement are essential to consolidate motor learning.

Clinical implications and injury prevention

Regarding injury prevention, evidence associates lower cadences with a higher incidence of overload injuries, especially tibial stress-related conditions (Luedke et al., 2016) [15]. Interventions based on increasing cadence have shown not only biomechanical but also clinical benefits, with high adherence among runners (Heiderscheit et al., 2011) [2]. In a rehabilitation context, Stockland et al. (2019) [16] showed that cadence can be maintained as a favorable variable, even with reduced weight support, reinforcing the therapeutic applicability of this strategy. Collectively, these findings support cadence retraining as a clinically relevant and accessible intervention, with positive outcomes across both biomechanical and clinical domains.

In line with these findings, a quasi-experimental study examined the combined effects of cadence modification, footwear adjustments, and orthotics on running biomechanics in recreational runners. The results showed that a moderate increase in cadence significantly reduced peak rearfoot impact forces and shortened stride length, reinforcing the potential of cadence modification to enhance running mechanics and reduce injury risk [17].

Furthermore, Menéndez et al. (2020) [18] conducted a systematic review focused on medial tibial stress syndrome (MTSS) in novice and recreational runners. Their review emphasized that modifying running technique, particularly through cadence adjustment, could alleviate the tibial load associated with MTSS. They identified intrinsic risk factors, such as increased hip internal rotation and excessive foot pronation, which could be mitigated through cadence adjustments, thereby reducing tibial stress and preventing injury.

Building on these findings, a 12-month gait retraining program incorporating cadence modification resulted in a 62% reduction in injury risk in novice runners [19], highlighting its value as an effective, low-cost, and accessible strategy for injury prevention across varied runner populations.

Discussion

The findings of this review confirm that a moderate increase (typically between 5% and 10%) in running cadence is an effective strategy for optimizing running biomechanics and reducing the risk of overload injuries. Reductions in impact forces and improvements in joint alignment were consistently observed and play a significant role in the prevention of common running-related injuries, such as patellofemoral pain and tibial stress fractures. Notably, contrary to earlier hypotheses, moderate increases in cadence do not appear to elevate energy expenditure and may even lead to subtle gains in running efficiency [13]. This reinforces the feasibility of cadence retraining as a valuable intervention for both clinical rehabilitation and performance-oriented training in recreational and competitive athletes.

A study included in this review conducted an eight-week intervention with an approximate 10% increase in cadence, which led most runners to transition from a rearfoot to a midfoot or forefoot strike pattern. This change was associated with a reduction in initial impact forces at foot contact, further supporting the protective role of cadence modification in preventing injury [20].

The primary biomechanical adaptations observed across the studies include reduced vertical ground reaction forces and loading rates, a shortened stride length [21], improved lower-limb joint alignment, particularly at the knee and hip, and a more favorable distribution of joint loading. These changes have been linked to a reduced incidence of overuse injuries, such as patellofemoral pain, tibial stress fractures, and medial tibial stress syndrome [15]. In line with these findings, de Souza Júnior et al. (2024) [22] demonstrated that gait retraining programs targeting both pain and lower-limb kinematics in runners with patellofemoral pain resulted in improved knee and hip alignment, further supporting the role of cadence modification in the prevention of knee-related injuries.

Neuromuscular adaptations also play an important role. Chumanov et al. (2012) [23] reported that a 10% cadence increase reduced peak hip adduction and abduction moments while increasing muscle activity during the late swing phase. This enhanced neuromuscular engagement likely contributes to a more protective landing posture by preparing the limb for foot-ground contact, which may be particularly beneficial for individuals with anterior knee pain. Importantly, these biomechanical and neuromuscular benefits were achieved without compromising running economy, underscoring the accessibility and cost-effectiveness of cadence retraining.

Moreover, individual responses to cadence modifications can vary significantly, influenced by factors such as technique, prior injury history, anthropometry, and running experience. Cadence adjustment should therefore be viewed as one element of a broader, personalized strategy that includes technical guidance, strength training, and load management.

The literature reviewed presents several limitations, including small sample sizes, methodological heterogeneity, and a predominance of surrogate biomechanical outcomes over clinically meaningful endpoints. Moderating variables such as fatigue, footwear type, and running surface remain underexplored, limiting the generalizability of current findings. Additionally, a notable limitation among several included studies is the lack of consistent control of running velocity during cadence manipulation protocols. Since changes in speed can independently influence ground reaction forces and joint loading, it becomes difficult to isolate the biomechanical effects of cadence modification alone. Future research should ensure consistent speed control across study conditions to allow clearer interpretation of cadence-specific effects. These gaps highlight the need for robust longitudinal studies with appropriate control groups to strengthen the evidence base in this field.

In summary, cadence retraining appears to be a promising intervention with both biomechanical and clinical benefits. When implemented under supervision and tailored to the runner’s individual profile, cadence retraining may significantly contribute to safer and more efficient running practices, enhancing both injury prevention and rehabilitation.

## Conclusions

This systematic review demonstrates that a moderate increase in running cadence (5-10% above spontaneous cadence, i.e., the self-selected number of steps per minute naturally adopted at a comfortable pace without external cues or intentional changes) induces beneficial biomechanical changes, including reduced vertical ground reaction forces, lower loading rates, shorter stride length, decreased vertical oscillation of the center of mass, and improved lower-limb joint alignment, particularly at the hip and knee, without compromising energy efficiency. These adaptations may contribute to a lower risk of common overuse injuries, such as patellofemoral pain, tibial stress fractures, and medial tibial stress syndrome. Importantly, cadence modification does not appear to negatively affect running economy and may, in some cases, enhance locomotor efficiency. The primary variables influenced by cadence retraining include impact attenuation, joint kinematics, temporal-spatial parameters, and strike pattern, all of which play a role in injury prevention.

Despite these promising results, the current evidence is limited by small sample sizes, short follow-up periods, and a predominance of biomechanical surrogate outcomes over long-term clinical endpoints. Future research should focus on well-designed, longitudinal studies with diverse runner populations, consistent control of running speed, and comprehensive assessment of both biomechanical and clinical outcomes to confirm cadence modification as an effective strategy for injury prevention and rehabilitation.
